# Congenital Circumferential Constriction Band of the Abdomen: A Case Report

**DOI:** 10.1155/2009/825174

**Published:** 2010-02-08

**Authors:** Kapil Gargh, Carol Sullivan, Hamish Laing, Sujoy Banerjee

**Affiliations:** ^1^Department of Neonatal Medicine, Singleton Hospital, ABM University Health Board, Swansea, SA2 8QA, UK; ^2^Welsh Centre for Burns & Plastic Surgery, ABM University Health Board, Swansea, SA6 6NL, UK

## Abstract

We report a case of congenital constriction band of abdomen associated with limb pseudarthrosis. The constriction band around the abdomen, though may cause initial difficulty with ventilation and parental distress, does not interfere with feeding, bowel movements, and growth. It heals spontaneously with supportive treatment though surgery may be needed in some cases.

## 1. Introduction

Though several hundred cases of congenital constriction band of the limbs have been described, that of abdomen is rare and only ten cases have been reported in the literature.

## 2. Case Report

**Figure 1:**
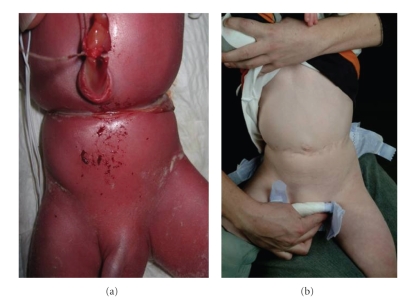
Appearance of the abdomen at birth (a) and at 2 years of age after surgical correction (b).

**Figure 2:**
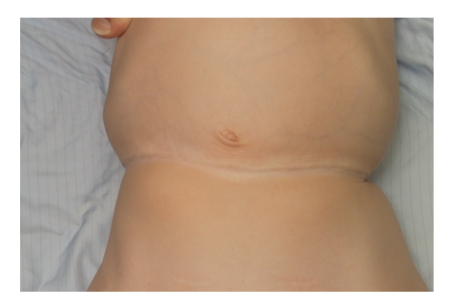
Appearance of the abdomen at 2 years of age before surgery.

**Figure 3:**
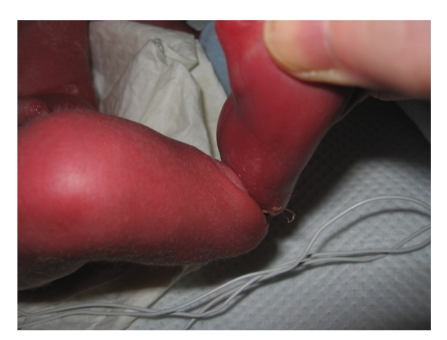
Appearance of pseudarthrosis of right leg at birth. A transtibial (below knee) amputation was later carried out.

**Figure 4:**
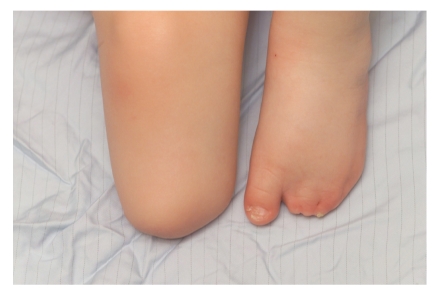
Appearance of right leg after amputation.

A 31-week preterm baby was noted at birth to have a deep circumferential constriction band of the abdomen (Figure 1). There was also a deep constriction ring involving the right leg that resulted in a pseudoarthrosis below knee (Figure 3). The clinical appearance of the abdomen was striking and initially appeared to interfere with breathing requiring ventilation on high pressures, but there were no mechanical problems affecting feeding or neurological pressure symptoms. In the absence of symptoms after the first few weeks, surgical correction using multiple Z-plasties was planned before school age. However, since he presented before that with abdominal pain, surgery was undertaken at 2 years of age (Figures 1 and 2). A transtibial (below knee) amputation was required for functional management of the right leg deformity (Figure 4).

## 3. Discussion

The overall reported prevalence of amniotic bands in the UK and Western Europe varies between 0.44 to 0.48 (per 10,000 births) [1]. Circumferential congenital constriction band of the abdomen is extremely rare [2–4]. Some common characteristics of the congenital constriction band of the abdomen have been described [5]. The constriction band follows a single segment that is higher on the back. The constriction ring may be located above or below the pelvic brim. The depth varies from a shallow groove to a deep gutter and usually extends only up to the first fascial layer. Histologically, the constriction bands are made of abundance of acellular fibrous tissue, or fibrous tissue containing fibroblasts, covered by squamous cells. This can make them inelastic and can produce a ligature effect. There may or may not be other associated congenital abnormalities. 

To the best of our knowledge, only 10 published cases of congenital constriction band involving the abdomen, with or without associated abnormalities, have been described (Table 1). 

Despite the alarming appearance, abdominal bands do not seem to cause mechanical problems [3]. Whilst the “hour glass” deformity can improve with time, plastic surgery may still be required as the abdominal contents expand or for cosmesis (Figure 1). 

## Figures and Tables

**Table 1:** Reported cases of congenital constriction band of the abdomen.

Author	Year	Location to pelvic brim	Sex	Other abnormalities
Brown et al.	1957	below	M	None
Schneider et al.	1976	below	F	Pilonodal sinus Cleft of soft palate
Evans	1973	above	F	None
Izumi et al.	1971	above	M	Band around 2 toes and club foot
Casaubon et al.	1983	above	F	None
Jones	1986	above	M	Limb defects
Bahadoran et al.	1997	above	F	None
Lin et al.	1999	above	M	None
Kim et al.	2007	above	F	Ring constriction of the left leg, absent hallux
Fawzy et al.	2008	below	F	Constriction bands on both calves, congenital amputations of toes
